# Are ethnic inequalities in COVID-19 outcomes mediated by occupation risk? Analyses of a 2-year record linked national cohort study in Scotland

**DOI:** 10.1093/eurpub/ckaf025

**Published:** 2025-03-05

**Authors:** Eliud Kibuchi, Sarah Amele, Ronan McCabe, Evangelia Demou, Alastair H Leyland, Colin R Simpson, Ting Shi, Patricia Irizar, Laia Becares, Aziz Sheikh, Anna Pearce, Srinivasa V Katikireddi

**Affiliations:** MRC/CSO Social & Public Health Sciences Unit, University of Glasgow, Glasgow, United Kingdom; MRC/CSO Social & Public Health Sciences Unit, University of Glasgow, Glasgow, United Kingdom; MRC/CSO Social & Public Health Sciences Unit, University of Glasgow, Glasgow, United Kingdom; MRC/CSO Social & Public Health Sciences Unit, University of Glasgow, Glasgow, United Kingdom; MRC/CSO Social & Public Health Sciences Unit, University of Glasgow, Glasgow, United Kingdom; Usher Institute, The University of Edinburgh, Edinburgh, United Kingdom; School of Health, Wellington Faculty of Health, Victoria University of Wellington, Wellington, New Zealand; Usher Institute, The University of Edinburgh, Edinburgh, United Kingdom; Department of Sociology, School of Social Sciences, University of Manchester, Manchester, United Kingdom; Department of Global Health & Medicine, King’s College London, London, United Kingdom; Usher Institute, The University of Edinburgh, Edinburgh, United Kingdom; MRC/CSO Social & Public Health Sciences Unit, University of Glasgow, Glasgow, United Kingdom; MRC/CSO Social & Public Health Sciences Unit, University of Glasgow, Glasgow, United Kingdom

## Abstract

This study investigated the extent to which ethnic inequalities in severe COVID-19 (i.e. hospitalization or deaths) are mediated through occupational risk differences. We used a population-based cohort study linking the 2011 Scottish Census to health records. We included all individuals aged 30–64 years and living in Scotland on 1 March 2020. The study period was from 1 March 2020 to 17 April 2022. Self-reported ethnicity was taken from the Census. We derived occupational risk of SARS-COV-2 infection using the 3-digit Standard Occupational Classification (SOC2010). We estimated hazard ratios (HRs) of total effects and controlled direct effects of ethnicity on severe COVID-19 mediated by occupational risk using marginal structural Cox models and subsequent proportional change. For aggregated ethnic groups, Non-White groups experienced a higher risk of severe COVID-19 (HR 1.6; 95% CI 1.4–1.8) compared to White group (all White ethnic groups) which increased to (1.7; 1.4–2.1) after accounting for occupational risk, representing a 6.0% change. For disaggregated ethnic groups, risks for South Asian (2.0; 1.8–2.3), African, Caribbean, or Black (1.3; 0.9–1.7) and Other ethnic groups (1.1; 0.9–1.3) were higher compared to White Scottish. After accounting for occupational risk, estimated risk of severe COVID-19 remained elevated for South Asian (1.8; 1.2–2.3), African Caribbean or Black (1.4; 0.8–2.1) and Other ethnic group (1.7; 1.1–2.3) representing a reduction of 11.8% and increases of 16.4% and 59.0%, respectively. Our findings suggest that ethnic inequalities in severe COVID-19 were impacted by differences in occupational risk.

## Introduction

Minority ethnic groups had disproportionate risk of SARS-CoV-2 infections and severe COVID-19 outcomes (hospitalization and deaths) in Scotland [1]. There is also well-documented evidence suggesting an association between occupation and the risk of SARS-CoV-2 transmission in UK [2–4]. However, little is known about the extent to which occupation explains, or mediates, ethnic inequalities in COVID-19 outcomes.

Current evidence suggests that determinants of occupational risk of SARS-CoV-2 infection depend on the number, frequency, duration, and proximity of infected individuals [5, 6]. Across the UK, occupations that were categorized as essential workers such as healthcare, education, and social care had particularly high SARS-CoV-2 infection rates [7–9]. This was mostly driven by an increased risk of exposure to SARS-CoV-2 infection [10] and close proximity to others since it was not possible for them to work from home [11]. For example, in 2020, essential workers in England and Wales had consistently higher all-cause monthly excess mortality of 50% or more, compared to other occupations, with healthcare workers affected worse compared to education and social care [4].

The UK minority ethnic groups (which are often categorized as Black, Asian, Mixed, and Other by the national statistical agency) are disproportionately represented in health and social care sectors at 14% compared to the average workforce of 12% [12]; making them potentially more susceptible to SARS-CoV-2 infections [2, 13] and subsequent severe COVID-19. This is further exacerbated by structural and institutional racism faced by minority ethnic groups which often leads to discriminatory tendencies such as being provided with inadequate personal protective equipment (PPE) [14, 15]. Considering the established associations between severe COVID-19 and both ethnicity and occupation, we can hypothesize that occupation mediates the relationship between ethnicity and severe COVID-19 outcomes. Given the substantial heterogeneity between minority ethnic groups, such mediation is likely to differ across ethnic groups. However, the occupation pathway linking ethnicity to COVID-19 outcomes is likely to be confounded or modified by other social determinants, such as education, which cannot be controlled for using traditional multivariable adjustments [16].

Therefore, this study aimed to examine the extent to which the risk of severe COVID-19 across ethnic groups in Scotland is mediated by occupation risk using marginal structural Cox models, a class of causal mediation models which can account for these complex confounding structures.

## Methods

### Study design and population

Data from the Early Pandemic Evaluation and Enhanced Surveillance of COVID-19 (EAVE II) study [17] were linked to the 2011 Scottish Census [18]. For this study, we included all individuals who were alive, aged 30–64 years on 1st March 2020 (i.e. were of working age during Census 2011) and residents of Scotland [17, 18]. The study period was from March 1, 2020 (date of first COVID-19 case in Scotland) to 17 April 2022.

The EAVE-II study includes ∼99% of the Scottish population and comprises primary care, testing, vaccination, hospitalization, and mortality data [17]. These data are linked together using the Community Health Index (CHI), a unique numeric identifier used by NHS Scotland within health records, which records registrations and de-registration with primary and secondary care services [18]. SARS-CoV-2 testing data were taken from the Electronic Communication of Surveillance in Scotland, morbidity and mortality data from Scottish Morbidity Record (SMR) and National Records of Scotland (NRS) datasets, respectively.

The 2011 Scottish Census took place on 27 March, and achieved an estimated response rate of 94% among an estimated population of 5.3 million individuals [1]. Ethnic classification was based on 16 groups, with the Non White ethnic groups constituting 3.4% of the Scottish population. The approximate linkage rate of 2011 Census to the EAVE-II study datasets was 94% [1]. We restricted our analysis to those individuals who were aged 30–64 years as of 1st March 2020, since they were of working age both in 2020 and 2011 when occupation data were collected.

### Outcome

The primary outcome was COVID-19 related hospitalization or death, combined to represent severe COVID-19. A COVID-19 related hospitalization was defined based on the International Classification of Diseases (ICD) 10 code (U07.1 and U07.2) listed in any diagnostic position or a hospitalization where the individual had a positive reverse transcription polymerase chain reaction (RT-PCR) test for SARS-CoV-2 in the 28 days prior to admission [19]. A COVID-19 related death was defined as either a death where U07.1 and U07.2 were recorded as the primary or secondary causes of death, or any death where the individual had a positive RT-PCR test for SARS-CoV-2 infection in the 28 days prior to death.

### Exposure

Self-reported ethnicity was obtained from the 2011 Scottish Census. We aggregated 16 categories into two different ethnic classifications to counter the low number of COVID-19 hospitalization and deaths: (i) White vs Non White, and (ii) White Scottish, Other White British or Irish, Other White, South Asian, African or Caribbean or Black, and Other (Supplementary Table S1).

### Mediator

Occupation risk was the mediating pathway considered. We classified occupation into four categories based on the potential risk of exposure to SARS-CoV-2: Low, medium, high, and economically inactive, based on the 2011 Scottish Census 3-digit Standard Occupational Classification (SOC2010). Assignment of SOC codes to each risk level were done independently by five authors (E.K., R.M., A.P., E.D., and S.V.K.) by considering the reported COVID-19 risks by occupations and workplaces in UK [9]. Where there was no clear consensus, authors held discussions and agreed on the appropriate risk level. The list of occupations categorized into different risk levels are included in Supplementary Table S2.

### Confounders

**Figure 1.**
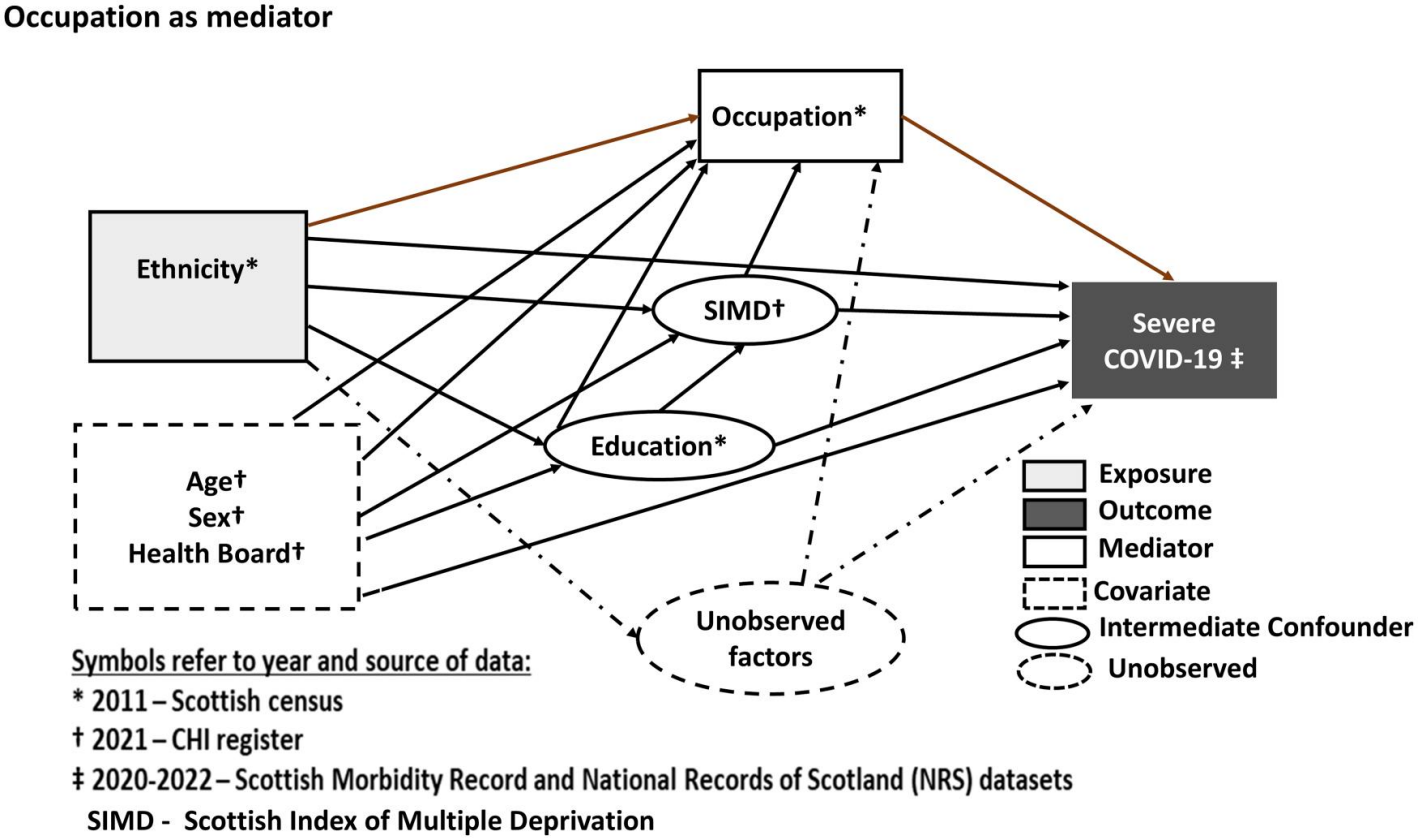
DAG summarizing the relationship between ethnicity, occupation, confounders, and COVID-19 outcomes.

Covariates included were sex (male vs female), age (continuous: 30–64 years), and health board (14 regions), all measured in 2020, taken from health records. Intermediate confounders were Scottish Index of Multiple Deprivation quintiles (1 = most deprived to 5 = least deprived) measured in 2020 (from health records), and education (non-degree and degree) measured in 2011 (in the Census). The hypothesized causal structure between ethnicity, occupation, confounders, and severe COVID-19 is presented in Fig. 1.

For sensitivity analyses, we also considered additional intermediate confounders: housing tenure (i.e. owned outright, owned with mortgage, social rented, private rented, and communal established), number of cars (i.e. 0,1, 2, and 3) and hours worked (i.e. 0, 1, 2, 3, 4); these were all measured in 2011. These intermediate confounders are both marginally and conditionally related with other variables in the directed acyclic graph (DAG) and the resulting sensitivity weights will be used to explore the extent at which they influence estimated effects. The causal structure for this is presented in Supplementary Fig. S1.

### Statistical analysis

We first described (with frequencies and percentages) the analysis sample.

We used marginal structural models (MSM) to estimate total effect (TE)—parameter estimate for ethnicity when severe COVID-19 is regressed on ethnicity and controlled direct effect (CDE)—parameter estimate for the ethnicity variable when severe COVID-19 is regressed on ethnicity and occupation [20–22]. We created two sets of inverse probability weights (IPW): (i) an exposure (i.e. ethnicity) IPW which accounts for covariates to create a pseudo population in which measured covariates are not associated with exposure, (ii) a mediator (i.e. occupation) IPW and this addresses the mediator-outcome relationship. For binary exposures (i.e. White vs Non White) we used logistic regression to estimate the IPW while for multicategory exposure and mediator variables, we used multinomial regression. Specifically, to estimate exposure weights we adjusted for sex, age, and health board. For mediator weights, we controlled for ethnicity, sex, age, health board, area deprivation, and education. Additionally, we included tenure and number of cars in estimating mediator weights for sensitivity weights. To improve precision of the estimated TE and CDE, extreme IPW values were truncated (i.e. stabilized) to have a mean of near one and a moderate range [23]. This is despite the possibility of increasing residual confounding due to truncation [21, 23]. Truncated exposure and mediator weights were then multiplied to obtain a final weight. Summary of weights for aggregated and disaggregated ethnicities are presented in Supplementary Tables S3 and S4.

TE and CDE were estimated using Cox proportional hazard (PH) regression [24], adjusting only for covariates for doubly robustness [25]. Intermediate confounders were not adjusted for as they lie on the causal pathway between ethnicity and severe COVID-19. To estimate TE, the model was weighted with the exposure weights only, while the final weights were used in CDE model, which also adjusted for the mediator. The TE and CDE were estimated under the following assumptions: (i) positivity (i.e. data were available for all conditions), (ii) no-unmeasured exposure-outcome or mediator-outcome confounding, (iii) consistency, and (iv) treatment variation irrelevance [22, 23, 26]. Details about these assumptions can be found in [22, 27].

We also computed proportion change (PC) of the ethnicity effects on severe COVID-19 if differences in occupation risk were eliminated by dividing the difference between TE and CDE by TE (i.e. $\left(\mathrm{TE}-\mathrm{CDE}\right)/\mathrm{TE}-1$) on the risk difference scale. We used a Monte Carlo approach to estimate confidence intervals for the PC. This involved sampling 1000 times from the distribution of the relative parameters (coefficients and variances/covariances) to derive the PC measure and taking the 2.5 and 97.5 percentiles of the distribution as its lower and upper confidence intervals, respectively. All analyses were done using R Statistical software (version 4.2.0) [28] with exposure and mediator IPW estimated using the “weightit” R package [29].

## Results

Table 1 shows the baseline characteristics of 2 064 982 individuals aged 30–64 years as of 1st March 2020 by ethnicity. For aggregated ethnicity, the White group were the majority (96.6%) with only 3.4% being Non White. For disaggregated ethnicity, White Scottish (84.6%) were the majority, followed by White British or Irish (8.6%), Other White (3.4%), South Asian (1.5%), Other (1.3%), and African, Caribbean or Black (0.5%). The proportion of Non White (0.7%) experiencing COVID-19 hospitalization or death was higher than White (0.5%) ethnicity. For the disaggregated ethnicity variable, the proportion experiencing severe COVID-19 was highest for the South Asian (0.9%), followed by White Scottish and African, Caribbean, or Black (0.6%), Other (0.5%), and lowest among White British or Irish and Other White (0.3%) ethnicities.

**Table 1.** Descriptive statistics by aggregated and disaggregated ethnicity

		Aggregated ethnicity		Disaggregated ethnicity						Total
		White	Non-White	White Scottish	White British or Irish	Other White	South Asian	African, Caribbean, or Black	Other	
		Frequency (%)	Frequency (%)	Frequency (%)	Frequency (%)	Frequency (%)	Frequency (%)	Frequency (%)	Frequency (%)	Frequency (%)
COVID-19 hospitalization or deaths		10 572 (0.5)	483 (0.7)	9741 (0.6)	597 (0.3)	234 (0.3)	288 (0.9)	67 (0.6)	128 (0.5)	11055 (0.5)
Occupation risk	Low	885 026 (44.4)	19 658 (28.2)	775 142 (44.4)	85 199 (47.8)	24 685 (35.6)	8536 (27.0)	3362 (31.7)	7760 (28.1)	904 684 (43.8)
	medium	680 058 (34.1)	27 515 (39.4)	597 964 (34.2)	49 517 (27.8)	32 577 (46.9)	13 353 (42.2)	3229 (30.5)	10 933 (39.6)	707 573 (34.3)
	High	375 451 (18.8)	12 848 (18.4)	325 120 (18.6)	40 610 (22.8)	9721 (14.0)	4947 (15.6)	2886 (27.2)	5015 (18.2)	388 299 (18.8)
	Economically inactive	54 617 (2.7)	9809 (14.0)	49 224 (2.8)	2945 (1.7)	2448 (3.5)	4808 (15.2)	1124 (10.6)	3877 (14.1)	64 426 (3.1)
Sex	Male	962 477 (48.2)	34 766 (49.8)	842 408 (48.2)	88 225 (49.5)	31 844 (45.9)	16 424 (51.9)	5507 (51.9)	12 835 (46.5)	99 7243 (48.3)
	Female	103 2675 (51.8)	35 064 (50.2)	905 042 (51.8)	90 046 (50.5)	37 587 (54.1)	15 220 (48.1)	5094 (48.1)	14 750 (53.5)	1 067 739 (51.7)
Age	[Mean (SD)]	48.20 (9.99)	44.97 (9.19)	48.26 (10.05)	49.33 (9.46)	43.92 (8.72)	44.89 (9.21)	44.91 (8.38)	45.07 (9.45)	48.09 (9.98)
Health Board	Ayrshire and Arran	145 847 (7.3)	1754 (2.5)	135 581 (7.8)	8538 (4.8)	1728 (2.5)	637 (2.0)	197 (1.9)	920 (3.3)	147 601 (7.1)
	Borders	40 342 (2.0)	435 (0.6)	32 737 (1.9)	6276 (3.5)	1329 (1.9)	115 (0.4)	70 (0.7)	250 (0.9)	40 777 (2.0)
	Dumfries and Galloway	55 144 (2.8)	686 (1.0)	45 993 (2.6)	8136 (4.6)	1015 (1.5)	206 (0.7)	108 (1.0)	372 (1.3)	55 830 (2.7)
	Forth Valley	109 011 (5.5)	1985 (2.8)	97 912 (5.6)	8511 (4.8)	2588 (3.7)	1006 (3.2)	226 (2.1)	753 (2.7)	110 996 (5.4)
	Grampian	221 820 (11.1)	7527 (10.8)	184 216 (10.5)	26 541 (14.9)	11 063 (15.9)	2273 (7.2)	1918 (18.1)	3336 (12.1)	229 347 (11.1)
	Highland	113 351 (5.7)	1638 (2.3)	92 249 (5.3)	16 576 (9.3)	4526 (6.5)	458 (1.4)	246 (2.3)	934 (3.4)	114 989 (5.6)
	Lothian	313 044 (15.7)	15 009 (21.5)	253 954 (14.5)	38 866 (21.8)	20 224 (29.1)	5960 (18.8)	2322 (21.9)	6727 (24.4)	328 053 (15.9)
	Orkney	7849 (0.4)	60 (0.1)	6375 (0.4)	1331 (0.7)	143 (0.2)	*	*	*	7909 (0.4)
	Shetland	8516 (0.4)	130 (0.2)	7041 (0.4)	1244 (0.7)	231 (0.3)	*	*	*	8646 (0.4)
	Western Isles	9669 (0.5)	98 (0.1)	8518 (0.5)	988 (0.6)	163 (0.2)	*	*	*	9767 (0.5)
	Fife	134 891 (6.8)	2726 (3.9)	119 617 (6.8)	11 726 (6.6)	3548 (5.1)	1156 (3.7)	376 (3.5)	1194 (4.3)	137 617 (6.7)
	Tayside	146 244 (7.3)	4066 (5.8)	127 678 (7.3)	13 021 (7.3)	5545 (8.0)	1787 (5.6)	529 (5.0)	1750 (6.3)	15 0310 (7.3)
	Greater Glasgow and Clyde	433 240 (21.7)	28 396 (40.7)	394 679 (22.6)	25 427 (14.3)	13 134 (18.9)	15 001 (47.4)	3965 (37.4)	9430 (34.2)	461 636 (22.4)
	Lanarkshire	256 184 (12.8)	5320 (7.6)	240 900 (13.8)	11 090 (6.2)	4194 (6.0)	2963 (9.4)	618 (5.8)	1739 (6.3)	261 504 (12.7)
Area deprivation	Most deprived	394 294 (19.8)	14 247 (20.4)	360 518 (20.6)	17 282 (9.7)	16 494 (23.8)	5148 (16.3)	3828 (36.1)	5271 (19.1)	408 541 (19.8)
	2	402 469 (20.2)	12 641 (18.1)	362 128 (20.7)	25 539 (14.3)	14 802 (21.3)	5895 (18.6)	2049 (19.3)	4697 (17.0)	415 110 (20.1)
	3	402 576 (20.2)	112 34 (16.1)	348 900 (20.0)	41 096 (23.1)	12 580 (18.1)	5226 (16.5)	1501 (14.2)	4507 (16.3)	413 810 (20.0)
	4	408 073 (20.5)	14 327 (20.5)	350 143 (20.0)	45 566 (25.6)	12 364 (17.8)	7242 (22.9)	1560 (14.7)	5525 (20.0)	422 400 (20.5)
	Least (deprived	387 740 (19.4)	17 381 (24.9)	325 761 (18.6)	48 788 (27.4)	13 191 (19.0)	8133 (25.7)	1663 (15.7)	7585 (27.5)	40 5121 (19.6)
Education	Non degree	139 6553 (70.0)	34 799 (49.8)	127 6926 (73.1)	87 113 (48.9)	32 514 (46.8)	16 955 (53.6)	4658 (43.9)	13 186 (47.8)	1 431 352 (69.3)
	degree	598 599 (30.0)	35 031 (50.2)	470 524 (26.9)	91 158 (51.1)	36 917 (53.2)	14 689 (46.4)	5943 (56.1)	14 399 (52.2)	633 630 (30.7)
Tenure	Owned (outright)	269 476 (13.5)	8923 (12.8)	238 141 (13.6)	27 080 (15.2)	4255 (6.1)	4612 (14.6)	411 (3.9)	3900 (14.1)	278 399 (13.5)
	Owned (mortgage)	1 080 413 (54.2)	31 143 (44.6)	960 941 (55.0)	99 236 (55.7)	20 236 (29.1)	17 148 (54.2)	2783 (26.3)	11 212 (40.6)	1 111 556 (53.8)
	Social rented	387 571 (19.4)	10 571 (15.1)	356 576 (20.4)	17 477 (9.8)	13 518 (19.5)	2679 (8.5)	3842 (36.2)	4050 (14.7)	398 142 (19.3)
	Private rented	246 394 (12.3)	17 436 (25.0)	183 548 (10.5)	32 973 (18.5)	29 873 (43.0)	6865 (21.7)	3188 (30.1)	7383 (26.8)	263 830 (12.8)
	Communal established	11 298 (0.6)	1757 (2.5)	8244 (0.5)	1505 (0.8)	1549 (2.2)	340 (1.1)	377 (3.6)	1040 (3.8)	13 055 (0.6)
Number of cars	0	339 450 (17.0)	16 613 (23.8)	297 281 (17.0)	23 649 (13.3)	18 520 (26.7)	5148 (16.3)	4192 (39.5)	7273 (26.4)	356 063 (17.2)
	1	748 146 (37.5)	26 516 (38.0)	649 109 (37.1)	66 379 (37.2)	32 658 (47.0)	11 291 (35.7)	4054 (38.2)	11 171 (40.5)	774 662 (37.5)
	2	896 258 (44.9)	24 944 (35.7)	792 816 (45.4)	86 738 (48.7)	16 704 (24.1)	14 865 (47.0)	1978 (18.7)	8101 (29.4)	921 202 (44.6)
	3	11 298 (0.6)	1757 (2.5)	8244 (0.5)	1505 (0.8)	1549 (2.2)	340 (1.1)	377 (3.6)	1040 (3.8)	13 055 (0.6)
Hours worked	Does not apply	54 617 (2.7)	9809 (14.0)	49 224 (2.8)	2945 (1.7)	2448 (3.5)	4808 (15.2)	1124 (10.6)	3877 (14.1)	64 426 (3.1)
	Part-time (15 h or less)	80 992 (4.1)	4045 (5.8)	68 485 (3.9)	9009 (5.1)	3498 (5.0)	1589 (5.0)	639 (6.0)	1817 (6.6)	85 037 (4.1)
	Part-time (16–30 h)	96 184 (4.8)	4388 (6.3)	85 818 (4.9)	7111 (4.0)	3255 (4.7)	2243 (7.1)	537 (5.1)	1608 (5.8)	100 572 (4.9)
	Full-time (31–48 h)	1 549 775 (77.7)	45 190 (64.7)	136 1471 (77.9)	134 575 (75.5)	53 729 (77.4)	19 702 (62.3)	7659 (72.2)	17 829 (64.6)	1 594 965 (77.2)
	Full-time (49 h or more hours)	213 584 (10.7)	6398 (9.2)	182 452 (10.4)	24 631 (13.8)	6501 (9.4)	3302 (10.4)	642 (6.1)	2454 (8.9)	219 982 (10.7)
Total		1 995 152 (96.6)	69 830 (3.4)	1 747 450 (84.6)	178 271 (8.6)	69 431 (3.4)	31 644 (1.5)	10 601 (0.5)	27 585 (1.3)	2 064 982

*Low numbers not allowable for disclosure release.

Most individuals in the White group were in low risk occupations (44.4%) compared to 28.2% in the Non White group, whereas 14% in the Non White group were economically inactive compared to 2.8% in the White group. There were similar distributions in the medium and high risk categories. For disaggregated ethnicity, the proportion of individuals engaged in high risk occupations was highest for African, Caribbean, or Black (27.2%), followed by White British or Irish (22.8%), White Scottish (18.6%), Other (18.2%), South Asian (15.2%), and Other White (14.0%). Economic inactivity was highest among South Asian (15.2%), followed by Other (14.1%), African, Caribbean, or Black (10.6%), Other White (3.5%), White Scottish (2.8%), and Other White British or Irish (1.7%).

Table 2 shows the effect estimates (TE, CDE and PC) for both aggregated and disaggregated ethnicities with corresponding summary statistics of IPWs presented in Supplementary Tables S5 and S6. For aggregated ethnicity, the Non White ethnic group had 60% higher risk of COVID-19 hospitalization or deaths TE: hazard ratio (HR 1.6; 95% CI 1.4–1.8) compared to the White group. After accounting for occupational risk, the TE increased slightly to (1.7; 1.4–2.1). This corresponds to an estimated increase in ethnic inequalities in COVID-19 hospitalization or death of 6% if differences in occupational risk exposure were eliminated. Sensitivity analyses conducted to explore inclusion of other intermediate confounders (i.e. tenure and number of cars) (Supplementary Table S7) were similar to those obtained in Table 2. We excluded the variable “number of hours” in the model because it was highly correlated with occupational risk, and this resulted in multicollinearity. However, we observed an estimated reduction in severe COVID-19 by 5% for the Non White ethnic group in models not adjusted for confounders to achieve double robustness (Supplementary Table S8). This demonstrates the necessity of undertaking doubly robust analysis to obtain unbiased estimates of ethnicity.

**Table 2.** Total effects (TE), controlled direct effects (CDE), and percentage change (PR) in relative inequalities in COVID 19 hospitalization or death according to both aggregated and disaggregated ethnicity if differences in occupation risk were eliminated

Variable	Category (reference)	TEs (Model 1)^a^ HR (95% CI)	CDEs (Model 2)^b^ HR (95% CI)	Percentage change Estimate (95% CI)
Aggregated ethnicity	White (ref)	1.00	1.00	
	Non-White	1.57 (1.45–1.76)	1.69 (1.35–2.13)	−6.12 (−6.21 to 6.03)
Disaggregated ethnicity	White Scottish (Ref)	1.00	1.00	
	White British or Irish	0.69 (0.63–0.75)	0.73 (0.62–0.84)	−5.70 (−5.12 to 0.29)
	Other White	0.86 (0.75–0.97)	0.68 (0.50–0.86)	19.00 (18.04 to 19.90)
	South Asian	2.02 (1.75–2.29)	1.75 (1.19–2.31)	11.78 (10.70 to 13.80)
	African, Caribbean, or Black	1.33 (0.93–1.73)	1.44 (0.82–2.06)	−16.39 (−19.00 to −13.80)
	Other	1.09 (0.85–1.33)	1.68 (1.10–2.26)	−59.01 (−31.31 to −56.71)

a Adjusted for confounders (i.e. age, sex, and health board) for doubly robustness.

b Adjusted for occupation risk and confounders (i.e. age, sex, and health board) for doubly robustness.

For disaggregated ethnicity, Table 2 shows that the White British or Irish and Other White ethnic groups were 31% and 14% less likely to experience severe COVID-19 compared to White Scottish, respectively. After accounting for occupation, the risk for White British or Irish increased slightly to (0.7; 0.6–0.8) representing an increase of 5.7%; and decreased to (0.7; 0.5–0.9) for Other White accounting for 19.0% reduction. On the other hand, South Asian (2.0; 1.8–2.3) and African, Caribbean, or Black (1.3; 0.9–1.7) had increased risk of COVID-19 hospitalization or death compared to White Scottish. Eliminating occupation risk differences, the South Asian (1.8; 1.2–2.3), African, Caribbean, or Black (1.4; 0.8–2.1) and Other ethnic group (1.1; 0.9–2.3) had an elevated risk of severe COVID-19 compared to White Scottish. This represented an increase in severe COVID-19 of 16.4% for African, Caribbean, or Black and 59.0% for Other ethnic group; and a reduction of 11.8% for South Asian if ethnicity differences in occupational risk were eliminated.

Sensitivity analyses that explored inclusion of other intermediate confounders (i.e. tenure and number of cars) and non-doubly robustness resulted in estimates similar to those obtained in Table 2 (Supplementary Tables S7 and S8).

## Discussion

Consistent with previous studies, we have found evidence of an increased risk of severe COVID-19 among minority ethnic groups compared to White majority [1, 30]. We found that this inequality increased (by 6%) once differences in occupation risk exposure were eliminated. For disaggregated ethnic groups, we estimated that up to 19% and 12% of ethnic inequalities in severe COVID-19 among Other White and South Asian groups would be reduced if differences in occupation risk were eliminated. On the other hand, we found that ethnic inequalities in severe COVID-19 among the White British or Irish, African, Caribbean, or Black and Other increased by 5%, 16%, and 59%, respectively, if all differences in occupational risk were eliminated. This study suggests that intervening on occupational risk exposure will lead to a reduction of severe COVID-19 among South Asian compared to White Scottish. It is important to note that interventions targeting occupational risks among disadvantaged ethnic groups (e.g. South Asian workers) may also result to better health outcomes for other groups employed in the same occupations.

The mixed mediating effect of occupational risk among minority ethnic groups in Scotland indicates the need for targeted rather than universal preventive interventions. These differences arise from the types of high-risk occupations commonly held by minority ethnic groups. For example, the 2011 Scottish Census data shows a larger proportion of African individuals were employed in caring, leisure and other service occupations; while Pakistani individuals (subset of South Asian group) were predominantly engaged in self-employed sales and customer service roles, each of which presents distinct exposures to COVID-19 risks [31]. Additionally, some minority ethnic groups in the UK, particularly Pakistani and Bangladeshi populations, are more prone to severe underlying health conditions and are more likely to live in multigenerational households, factors that may have compounded their risk of hospitalization or death from COVID-19 [32, 33].

This will help avoid a situation where certain occupational interventions may lead to an increase in ethnic inequalities among certain groups if applied universally. Therefore, targeted interventions such as providing PPE designed with ethnic minorities features on mind (e.g. facial dimension features in the case of face masks) [34], initiating structural changes aimed at promoting social justice and racial equity in workplaces [15, 35], increasing opportunities to work from home [11], and income security [36] can lead to reduced ethnic inequalities mediated by occupation. Moreover, since occupational risk differences were also observed among majority populations (e.g. White Scottish vs White British or Irish), improving working conditions for those worse affected such as South Asian group, could contribute to broader reductions in occupational risk-induced social inequalities in general population.

The fact we categorized occupations based on perceived average risk may have impacted the overall results since different occupations experienced varying levels of risks across different waves during the pandemic [3]. For example, healthcare workers experienced elevated risks at the start of the pandemic which reduced over time while education workers faced persistent risks throughout the pandemic [3]. Moreover, occupational risk of severe COVID-19 associated with each occupation varied across the pandemic due to different levels of exposure to SARS-CoV-2 infection based on lockdown measures at each time and PPE provided. We combined some minority ethnic groups into broad groups to overcome the issue of fewer or no COVID-19 related hospitalizations or deaths. However, this may have contributed to mixed risks of severe COVID-19 among minority ethnic groups because important differences exist within these ethnic categorizations [1]. Moreover, considering ethnicity is a complex social construct, grouping individuals into this broad ethnic groups may have masked important differences that would have allowed for more disaggregated estimations [37]. This explains why adjusting for occupational risk leads to a higher risk in some ethnic groups compared to others due to differential vulnerability of the COVID-19 among individual ethnic groups which complicates interpretation of the obtained result.

The analysis data only contained people aged 30–64 years who were enumerated during the 2011 Scottish Census and recorded in the CHI register as of 1st March 2020. Therefore, this analysis excluded everyone who was 29 years or younger as of March 2020, an estimated 6% of people who were living in Scotland but did not take part in 2011 Census, respondents who could not be linked to the EAVE-II data, and people who have emigrated to Scotland since 2011. Therefore, these results may not fully capture the extent to which occupational risks mediated ethnic inequalities in COVID-19 among minority ethnic groups. For example, newly immigrated individuals, who predominantly belong to minority ethnic groups, are more likely to work in high-risk exposure occupations [38]. Moreover, they often face structural barriers to accessing healthcare, such as language difficulties and limited eligibility for economic support benefits [38]. However, overall findings aligned with other studies demonstrating that minority ethnic groups and migrants were disproportionately represented in high-risk occupations leading to greater exposure and infection rates of COVID-19 compared to majority groups [39].

The assumptions that MSM models used in our analyses were correctly specified, and that consistency assumption holds (i.e. ethnicity and occupation have the same effect on severe COVID-19 for everyone, despite how everyone’s ethnicity and occupations were defined) are difficult to ascertain [40, 41]. We also assumed that individuals held the same occupations in 2020 as recorded in Census 2011. Therefore, any changes in occupations between 2011 and 2020 are likely to have led to biased estimates. Moreover, we assumed there were no differences in occupational risk in jobs within the same SOC code. This is not always the case. For example, both a surgeon and a veterinarian doctor are in the same healthcare professionals SOC code, but they experienced different risks of SARS-COV-2 exposure. Also, categorizations of occupations based on susceptibility to COVID-19 by authors may have substantially changed the results despite their efforts to reduce this by having each risk level classification done independently. In addition, confounders derived from Census data, such as education, may have changed over time, particularly among the youngest age groups which may have led to biased estimates. However, newer data are not available. Finally, use of IPW does not address unmeasured confounding. For example, unmeasured confounders such as clinical and lifestyle factors may potentially confound the association between ethnicity and COVID-19 risk.

Despite these limitations, this study highlights the potential role of occupation in shaping ethnic inequalities during COVID-19 pandemic. In summary, considering the level of occupational risk to COVID-19 increased the risk of severe COVID-19 among some minority ethnic groups, intervening on occupations with higher risk of exposure may reduce the ethnic inequalities in future pandemics.

## Supplementary Material

ckaf025_Supplementary_Data

## Data Availability

The data used in this study include sensitive category individual-level data. To prevent disclosure these data are not publicly available but are available for research purposes through successful application to NHS Scotland Public Benefit and Privacy Panel for Health and Social Care (HSC-PBPP) and NRS. Researchers interested in the data may contact the Electronic Data Research and Innovation Service (eDRIS) phs.edris@phs.scot
 Key points Ethnic inequalities increased by 6% among minority ethnic groups compared to White majority once differences in occupation risk exposure were eliminated. For disaggregated ethnicity, ethnic inequalities in severe COVID-19 among the White British or Irish, African, Caribbean, or Black and Other increased by 5%, 16%, and 59%, respectively, if all differences in occupational risk were eliminated. However, up to 19% and 12% of ethnic inequalities in severe COVID-19 among Other White and South Asian groups would be reduced if differences in occupation risk were eliminated. The mixed mediating effect of occupational risk among minority ethnic groups in Scotland indicates the need for targeted rather than universal preventive interventions.
