# Supplementary material for: Are ethnic inequalities in COVID-19 outcomes mediated by occupation risk? Analyses of a 2-year record linked national cohort study in Scotland
Source: Eur J Public Health. 2025 Mar 5;35(2):379–85. doi: 10.1093/eurpub/ckaf025 (PMC11967892; doi:10.1093/eurpub/ckaf025)
Supplement: ckaf025_Supplementary_Data [file ckaf025_supplementary_data.docx]

**Supplementary material: Are ethnic inequalities in COVID-19 outcomes mediated by occupation risk? Analyses of a two-year record linked national cohort study in Scotland.**

Table S1: Ethnicity classifications based in 2011 Scottish Census

| **Dis-aggregated ethnic groups in 2011 Scottish Census** | **Categorical ethnicity variable** | **Binary ethnicity variable** |
| --- | --- | --- |
| White Scottish | White Scottish | White |
| White Other British | White Other British or Irish |  |
| White Irish |  |  |
| White Gypsy/Traveller | Other White |  |
| White Polish |  |  |
| Other White |  |  |
| Pakistani, Pakistani Scottish or Pakistani British (referred to in main text as Pakistani) | South Asian | Non-White |
| Indian, Indian Scottish, or Indian British (referred to in main text as Indian) |  |  |
| Bangladeshi, Bangladeshi Scottish or Bangladeshi British (referred to in main text as Bangladeshi) |  |  |
| African, African Scottish, or African British (referred to in main text as African) | African, Caribbean, or Black |  |
| Caribbean, Caribbean Scottish or Caribbean British/Black, Black Scottish or Black British (referred to in main text as African or Black) |  |  |
| Arab, Arab Scottish, or Arab British (referred to in main text as Arab) | Other Ethnicity |  |
| Chinese, Chinese Scottish or Chinese British (referred to in main text as Chinese) |  |  |
| Other Asian |  |  |
| Mixed or Multiple Ethnicity |  |  |
| Other Ethnicity |  |  |

Table S2: List of occupations categorised into different COVID-19 risk levels.

| **3-digit SOC group** | **SOC Number** | **COVID-19 occupation risk** |
| --- | --- | --- |
| 111 111 'Chief Executives and Senior Officials' | 111 | Low |
| 112 112 'Production Managers and Directors' | 112 | Low |
| 113 113 'Functional Managers and Directors' | 113 | Low |
| 115 115 'Financial Institution Managers and Directors' | 115 | Low |
| 116 116 'Managers and Directors in Transport and Logistics' | 116 | Medium |
| 117 117 'Senior Officers in Protective Services' | 117 | Medium |
| 118 118 'Health and Social Services Managers and Directors' | 118 | Medium |
| 119 119 'Managers and Directors in Retail and Wholesale' | 119 | Medium |
| 121 121 'Managers and Proprietors in Agriculture Related Srvcs' | 121 | Low |
| 122 122 'Managers and Proprietors in Hsptlty and Leisure Srvcs' | 122 | Medium |
| 124 124 'Managers and Proprietors in Health and Care Services' | 124 | Medium |
| 125 125 'Managers and Proprietors in Other Services' | 125 | Medium |
| 211 211 'Natural and Social Science Professionals' | 211 | Low |
| 212 212 'Engineering Professionals' | 212 | Low |
| 213 213 'IT and Telecommunications Professionals' | 213 | Low |
| 214 214 'Conservation and Environment Professionals' | 214 | Low |
| 215 215 'Research and Development Managers ' | 215 | Low |
| 221 221 'Health Professionals' | 221 | High |
| 222 222 'Therapy Professionals' | 222 | High |
| 223 223 'Nursing and Midwifery Professionals' | 223 | High |
| 231 231 'Teaching and Educational Professionals' | 231 | High |
| 241 241 'Legal Professionals' | 241 | Low |
| 242 242 'Business, Research and Administrative Professionals' | 242 | Low |
| 243 243 'Architects, Town Planners and Surveyors' | 243 | Low |
| 244 244 'Welfare Professionals' | 244 | High |
| 245 245 'Librarians and Related Professionals' | 245 | Low |
| 246 246 'Quality and Regulatory Professionals' | 246 | Low |
| 247 247 'Media Professionals' | 247 | Low |
| 311 311 'Science, Engineering and Production Technicians' | 311 | Low |
| 312 312 'Draughtspersons and Related Architectural Technicians' | 312 | Low |
| 313 313 'Information Technology Technicians' | 313 | Low |
| 321 321 'Health Associate Professionals' | 321 | High |
| 323 323 'Welfare and Housing Associate Professionals' | 323 | High |
| 331 331 'Protective Service Occupations' | 331 | High |
| 341 341 'Artistic, Literary and Media Occupations' | 341 | Low |
| 342 342 'Design Occupations' | 342 | Low |
| 344 344 'Sports and Fitness Occupations' | 344 | Medium |
| 351 351 'Transport Associate Professionals' | 351 | Low |
| 352 352 'Legal Associate Professionals' | 352 | Low |
| 353 353 'Business, Finance and Related Associate Professionals' | 353 | Low |
| 354 354 'Sales, Marketing and Related Associate Professionals' | 354 | Low |
| 355 355 'Conservation and Environmental Assct Professionals' | 355 | Low |
| 356 356 'Public Services and Other Associate Professionals' | 356 | Low |
| 411 411 'Administrative Occupations: Gvrnmnt and Related Orgs' | 411 | Low |
| 412 412 'Administrative Occupations: Finance' | 412 | Low |
| 413 413 'Administrative Occupations: Records' | 413 | Low |
| 415 415 'Other Administrative Occupations' | 415 | Low |
| 416 416 'Administrative Occupations: Office Mngrs and Sprvsrs' | 416 | Low |
| 421 421 'Secretarial and Related Occupations' | 421 | Medium |
| 511 511 'Agricultural and Related Trades' | 511 | Low |
| 521 521 'Metal Forming, Welding and Related Trades' | 521 | Low |
| 522 522 'Metal Machining, Fitting and Instrument Making Trades' | 522 | Low |
| 523 523 'Vehicle Trades' | 523 | Low |
| 524 524 'Electrical and Electronic Trades' | 524 | Low |
| 525 525 'Skilled Metal, Electrical and Electronic Trds Sprvsrs' | 525 | Low |
| 531 531 'Construction and Building Trades' | 531 | Low |
| 532 532 'Building Finishing Trades' | 532 | Low |
| 533 533 'Construction and Building Trades Supervisors' | 533 | Low |
| 541 541 'Textiles and Garments Trades' | 541 | Low |
| 542 542 'Printing Trades' | 542 | Low |
| 543 543 'Food Preparation and Hospitality Trades' | 543 | Medium |
| 544 544 'Other Skilled Trades' | 544 | Low |
| 612 612 'Childcare and Related Personal Services' | 612 | High |
| 613 613 'Animal Care and Control Services' | 613 | Low |
| 614 614 'Caring Personal Services' | 614 | High |
| 621 621 'Leisure and Travel Services' | 621 | Medium |
| 622 622 'Hairdressers and Related Services' | 622 | Medium |
| 623 623 'Housekeeping and Related Services' | 623 | Medium |
| 624 624 'Cleaning and Housekeeping Managers and Supervisors' | 624 | Medium |
| 711 711 'Sales Assistants and Retail Cashiers' | 711 | Medium |
| 712 712 'Sales Related Occupations' | 712 | Medium |
| 713 713 'Sales Supervisors' | 713 | Medium |
| 721 721 'Customer Service Occupations' | 721 | Low |
| 722 722 'Customer Service Managers and Supervisors' | 722 | Low |
| 811 811 'Process Operatives' | 811 | Medium |
| 812 812 'Plant and Machine Operatives' | 812 | Low |
| 813 813 'Assemblers and Routine Operatives' | 813 | Low |
| 814 814 'Construction Operatives' | 814 | Low |
| 821 821 'Road Transport Drivers' | 821 | Medium |
| 822 822 'Mobile Machine Drivers and Operatives' | 822 | Low |
| 823 823 'Other Drivers and Transport Operatives' | 823 | Low |
| 911 911 'Elementary Agricultural Occupations' | 911 | Low |
| 912 912 'Elementary Construction Occupations' | 912 | Low |
| 913 913 'Elementary Process Plant Occupations' | 913 | Medium |
| 921 921 'Elementary Administration Occupations' | 921 | Medium |
| 923 923 'Elementary Cleaning Occupations' | 923 | Medium |
| 924 924 'Elementary Security Occupations' | 924 | Medium |
| 925 925 'Elementary Sales Occupations' | 925 | Medium |
| 926 926 'Elementary Storage Occupations' | 926 | Medium |
| 927 927 'Other Elementary Services Occupations' | 927 | Medium |

**
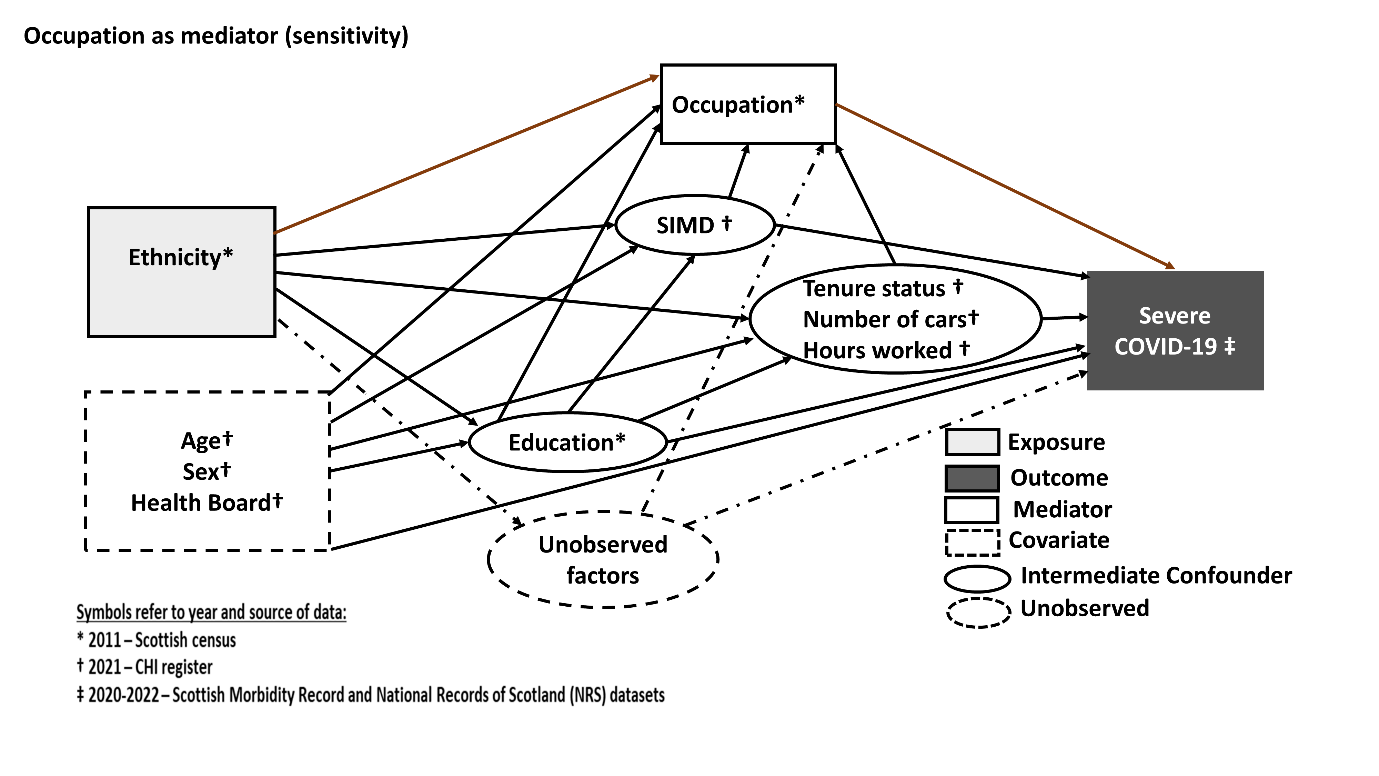
** Figure S1: Directed acyclic graph summarising the relationship between ethnicity, occupation (SOC3) and Covid-19 outcomes – sensitivity analysis.

Table S3: Distribution of inverse propensity weights (IPW) distribution for aggregated (White vs Non-White) ethnic groups.

| Category of IPW | Minimum | 1st quantile | Median | Mean | 3rd quantile | Maximum |
| --- | --- | --- | --- | --- | --- | --- |
| Exposure | 0.51 | 0.98 | 0.99 | 0.99 | 1.01 | 1.09 |
| Mediation | 0.34 | 0.69 | 0.84 | 0.99 | 1.13 | 3.12 |
| Final (Exposure * Mediation) | 0.17 | 0.69 | 0.84 | 0.97 | 1.13 | 3.4 |
| mediation (sensitivity) | 0.24 | 0.66 | 0.83 | 0.98 | 1.14 | 3.29 |
| final (Exposure * Mediation (sensitivity)) | 0.12 | 0.66 | 0.82 | 0.97 | 1.13 | 3.58 |

Table S4: Distribution of inverse propensity weights (IPW) distribution for disaggregated (White Scottish, White British or Irish, Other White, South Asian, African, Caribbean or Black and Other) ethnic groups.

| Category of IPW | Minimum | 1st quantile | Median | Mean | 3rd quantile | Maximum |
| --- | --- | --- | --- | --- | --- | --- |
| Exposure | 0.38 | 0.94 | 0.98 | 0.99 | 1.04 | 2.02 |
| Mediation | 0.33 | 0.69 | 0.84 | 0.99 | 1.12 | 3.17 |
| Final (Exposure * Mediation) | 0.13 | 0.67 | 0.82 | 0.98 | 1.14 | 6.41 |
| mediation (sensitivity) | 0.24 | 0.66 | 0.83 | 0.98 | 1.13 | 3.34 |
| final (Exposure * Mediation (sensitivity)) | 0.09 | 0.63 | 0.81 | 0.97 | 1.14 | 6.77 |

Table S5: Distribution of inverse propensity weights (IPW) distribution for aggregated (White vs Non-White) ethnic groups.

| Category of IPW | Minimum | 1st quantile | Median | Mean | 3rd quantile | Maximum |
| --- | --- | --- | --- | --- | --- | --- |
| Exposure | 0.51 | 0.98 | 0.99 | 0.99 | 1.01 | 1.09 |
| Mediation | 0.34 | 0.69 | 0.84 | 0.99 | 1.13 | 3.12 |
| Final (Exposure * Mediation) | 0.17 | 0.69 | 0.84 | 0.97 | 1.13 | 3.4 |
| mediation (sensitivity) | 0.24 | 0.66 | 0.83 | 0.98 | 1.14 | 3.29 |
| final (Exposure * Mediation (sensitivity)) | 0.12 | 0.66 | 0.82 | 0.97 | 1.13 | 3.58 |

Table S6: Distribution of inverse propensity weights (IPW) distribution for disaggregated (White Scottish, White British or Irish, Other White, South Asian, African, Caribbean or Black and Other) ethnic groups.

| Category of IPW | Minimum | 1st quantile | Median | Mean | 3rd quantile | Maximum |
| --- | --- | --- | --- | --- | --- | --- |
| Exposure | 0.38 | 0.94 | 0.98 | 0.99 | 1.04 | 2.02 |
| Mediation | 0.33 | 0.69 | 0.84 | 0.99 | 1.12 | 3.17 |
| Final (Exposure * Mediation) | 0.13 | 0.67 | 0.82 | 0.98 | 1.14 | 6.41 |
| mediation (sensitivity) | 0.24 | 0.66 | 0.83 | 0.98 | 1.13 | 3.34 |
| final (Exposure * Mediation (sensitivity)) | 0.09 | 0.63 | 0.81 | 0.97 | 1.14 | 6.77 |

Table S7: Total effects (TE) controlled direct effects (CDE), and percentage change (PC) in relative inequalities in COVID 19 hospitalisation or death according to both aggregated and disaggregated ethnicity if differences in occupation risk were eliminated using sensitivity weights.

| Variable | Category (reference) | Total Effects  (Model 1) ^a^  HR (95% CI) | Controlled Direct Effects (Model 2) ^b.^  HR (95% CI) | Percentage Change  Estimate (95% CI) |
| --- | --- | --- | --- | --- |
| Aggregated ethnicity | White (ref) | 1.00 | 1.00 |  |
|  | Non White | 1.57 (1.45-1.76) | 1.69(1.35 -2.13) | -6.12(-6.21—6.03) |
| Disaggregated ethnicity | White Scottish (Ref) | 1.00 | 1.00 |  |
|  | White British or Irish | 0.69 (0.05 - -8.21) | 0.73 (0.07 -4.33) | -5.70 (-5.12 – 0.29) |
|  | Other White | 0.86 (0.08 - -2.04) | 0.68 (0.16 -2.42) | 19.00 (18.04 – 19.90) |
|  | South Asian | 2.02 (0.07 - 1.34) | 1.75 (0.18 - 3.06) | 11.78 (10.70 – 13.80) |
|  | African, Caribbean, or Black | 1.33 (0.15 - 2.00) | 1.44 (0.32 - 1.13) | -16.39 ( -19.00 - -13.80) |
|  | Other | 1.09 (0.10 - 0.85) | 1.68 (0.20 - 2.54) | -59.01 (-31.31 – -56.71) |

^a^ Adjusted for confounders (i.e. age, sex, and health board) for doubly robustness

^b^ Adjusted for occupation risk and confounders (i.e., age, sex, and health board) for doubly robustness

Table S8: Total effects (TE), controlled direct effects (CDE), and percentage change (PC) in relative inequalities in COVID 19 hospitalisation or death according to both aggregated and disaggregated ethnicity if differences in occupation risk were eliminated (non-doubly robust)

| Variable | Category (reference) | Total Effects  (Model 1) ^a^  HR (95% CI) | Controlled Direct Effects (Model 2) ^b.^  HR (95% CI) | Percentage Change.  Estimate (95% CI) |
| --- | --- | --- | --- | --- |
| Aggregated ethnicity | White (ref) | 1.00 | 1.00 |  |
|  | Non White | 1.46 (1.33-160) | 1.38 (1.10 – 1.73) | 5.22 (5.14 – 5.30) |
| Disaggregated ethnicity | White Scottish (Ref) | 1.00 | 1.00 |  |
|  | White British or Irish | 0.68 (0.63 – 0.75) | 0.73 (0.63 – 0.85) | -7.56 (-8.14 – -6.98) |
|  | Other White | 0.79 (0.68 – 0.92) | 0.65 (0.48 – 0.89) | 17.29 (16.43 – 18.16) |
|  | South Asian | 1.96 (1.71- 2.24) | 1.45 (1.02 – 2.08) | 24.24 (23.33 – 25.16) |
|  | African, Caribbean, or Black | 1.24 (0.93 – 1.65) | 1.29 (0.69 – 2.43) | -10.79 ( -13.41 - -8.18) |
|  | Other | 1.04 (0.86 – 1.26) | 1.51 (1.01 – 2.25) | -59.19 (-52.37 – -48.01) |
